# Delineating circulating lymphocyte subsets in the transition from gout remission to recurrence

**DOI:** 10.3389/fimmu.2025.1540429

**Published:** 2025-04-17

**Authors:** Hanjie Yu, Ling Wang, Ling Qin, Hanqing Yu, Ran Hu, Zhenghu Jia, Hui Bao, Haichao Wang, Wen Xue, Yaxiang Song, Zhinan Yin, Ai Peng

**Affiliations:** ^1^ Center for Nephrology and Clinical Metabolomics and Division of Nephrology, Shanghai Tenth People’s Hospital, Tongji University School of Medicine, Shanghai, China; ^2^ Clinical Medicine Scientific and Technical Innovation Center, Shanghai Tenth People’s Hospital, Tongji University School of Medicine, Shanghai, China; ^3^ Guangdong Provincial Key Laboratory of Tumor Interventional Diagnosis and Treatment, Zhuhai Institute of Translational Medicine, Zhuhai People’s Hospital (Zhuhai Clinical Medical College of Jinan University), Jinan University, Zhuhai, China; ^4^ State Key Laboratory of Bioactive Molecules and Druggability Assessment, The Biomedical Translational Research Institute, Health Science Center (School of Medicine), Jinan University, Guangzhou, China; ^5^ Guangzhou Purui Biotechnology Co., Ltd., Research and Development Center, Guangzhou, Guangdong, China; ^6^ Key Laboratory of Viral Pathogenesis & Infection Prevention and Control (Jinan University), Ministry of Education, Guangzhou, China

**Keywords:** lymphocyte immunophenotype, gout remission, gout recurrence, predictive model, conversion pattern

## Abstract

**Objectives:**

Lymphocytes and their subsets are implicated in both the onset and remission of gout. However, the specific roles in gout recurrence and complete remission remain unclear. This study aimed to characterize lymphocyte immunophenotypes across different stages of gout and developed a predictive model for remission and recurrence of gout.

**Methods:**

Plasma levels of 75 lymphocyte immunophenotypes were determined using multiplex flow cytometry in patients with acute gout flare (AG, n=78), gout remission (RG, n=63), and healthy controls (NC, n=66). Lymphocyte immunophenotyping candidates and significant clinical parameters were subjected to LASSO regression for conducting a predictive model.

**Results:**

Significant variations in lymphocyte profiles were identified among the groups. A combination of T peripheral helper cells, virus-specific cytotoxic natural killer (NK) cells, inhibition of Vδ1 and Vδ2 cells, along with BMI, eGFR, hemoglobin, uric acid, distinguished RG from NC (AUC=0.934). Similarly, inhibition of Vδ2 cells, virus-specific cytotoxic NK cells, inactive and terminally differentiated virus-specific CD8^+^ T cells, plus hematological parameters, classified RG from AG (AUC = 0.814) and predicted gout recurrence in a one-year follow-up validation cohort (AUC = 0.724). Inhibition of Vδ2 cells and virus-infected specific cytotoxic NK cells are strongly associated with gout recurrence and complete remission.

**Conclusion:**

Significant alterations in lymphocyte immunophenotypes, notably the inhibition of Vδ2 cells and virus-infected specific cytotoxic NK cells during the transition from gout recurrence to complete remission, provide compelling evidence to enhance the clinical delineation of gout stages and propel mechanistic investigations into the progression of gout.

## 1 Introduction

Gout, a prevalent and complex form of arthritis, is triggered by the deposition of monosodium urate (MSU) crystals in joints and tissues, leading to systemic inflammation and tissue damage (1, 2). This condition is characterized by acute flares, periods of remission, and the potential recurrence after symptoms subside (3, 4).

Recently, one study proposed preliminary criteria for remission, including serum uric acid, acute attacks, gout tophi, pain, and overall patient assessment (5, 6). Another study using dual-source computed Tomography (CT) demonstrates that some crystal deposition may still be present in patients meeting aforementioned criteria (7). While preventing recurrent gout flares is a priority for improving outcomes, the conversion in disease patterns of gout remain poorly understood.

The role of immune cells, particularly through innate immunity mechanisms, has been recognized as pivotal in the pathogenesis of gout (3, 8). While the involvement of tissue macrophages and monocyte-derived macrophages in joints has been extensively studied, the transition of lymphocytes from gout remission to flare is still unclear (9–12). Previous studies highlight the role of various T lymphocytes in gouty arthritis, noting that T helper (Th)17, Th1, Th9, Th22, and γδT cells play an enhancing role in gout attacks, while Tregs and Th2 cells have a suppressive effect (10, 13). Our preliminary findings indicate significant immune cell dysregulation in patients with gout flare and remission. More importantly, even patients in remission exhibit T cell subtypes distribution markedly different from that of healthy individuals (13, 14).

Given the potential involvement of lymphocytes in the transition from gout remission to its recurrence, we characterize the circulating immunophenotypes of lymphocytes in the plasma across various stages of gout, and subsequently, based on their featured changes to develop a predictive model for complete remission and potential recurrence of gout.

## 2 Materials and methods

### 2.1 Participants

This prospective cohort study (NCT05522504) was conducted in accordance with the Declaration of Helsinki and approved by the Institutional Review Board, Medical Ethics Committee of Shanghai Tenth People’s Hospital of Tongji University (SHSY-IEC-4.1/21-246/01). Informed written consent was obtained from all participants.

Participants experiencing a gout flare were included if they met the following criteria: 1) aged 18–80 years; 2) onset of acute gout flare within the past 24 hours, meeting the 2015 American College of Rheumatology (ACR)/European League Against Rheumatism (EULAR) classification criteria (15); 3) no treatment received in the three days before blood sampling; 4) detection of MSU crystals by B-mode ultrasonography or dual-source dual-energy CT.

Participants in gout remission were included if they met the following criteria: 1) aged 18–80 years; 2) previous gout diagnosis according to the ACR Board of Directors and the EULAR Executive Committee (15); 3) absence of clinical manifestations—redness, swelling, heat, and pain in local joints or bursae—for at least three months since the last gout flare; 4) normal erythrocyte sedimentation rate and C-reactive protein levels over the past three months (5); detection of MSU crystals by B-mode ultrasonography or dual-source dual-energy CT.

Exclusion criteria for gout patients were: (1) unwillingness to participate; (2) acute infection or treatment for infections (viruses, bacteria, fungi, rickettsiae, spirochetes, or parasites) in the last three months; (3) receipt of any vaccines, including rabies, influenza, or corona virus disease 2019 (COVID-19) vaccines, in the last three months; (4) blood transfusion, significant blood loss, donation of 200 mL or more, receipt of transfusions, or use of blood products in the past three months; (5) immunodeficiency or immunosuppression; (6) use of corticosteroids or indomethacin in the past three months; (7) septic arthritis or other joint diseases (e.g., rheumatoid arthritis); (8) renal dysfunction (estimated glomerular filtration rate <30 mL/min/1.73 m²); (9) other conditions that may elevate serum uric acid levels.

The inclusion criteria for the control group were as previously described (14): (1) aged 18–80 years; (2) no self-reported or clinically diagnosed history of hyperuricemia or gout; (3) no treatment received in the three days before blood sampling. The exclusion criteria were identical to those for the gout patients. The three groups were fully age-matched (statistical significance >0.1).

### 2.2 Blood sample collection

Peripheral venous blood samples were collected from gout patients. Two milliliters of venous blood from each patient were added to vacuum tubes containing anticoagulant ethylenediaminetetraacetic acid (EDTA) solution for peripheral blood cell counts and biochemical examinations. An additional four milliliters of venous blood were collected in vacuum tubes for flow cytometry. Peripheral blood mononuclear cells (PBMCs) were isolated using Ficoll density gradient centrifugation (16). The flow cytometry and blood biochemical analyses were performed on the same blood samples. All examinations were conducted at the Department of Laboratory Medicine of Shanghai Tenth People’s Hospital.

### 2.3 Flow cytometry analysis

Centrifuge antibody briefly (3000 rpm, 4°C) and prepare the antibody working solution according to the antibody manual (the overall system antibody ratio is 1:10), gently vortex, and set on ice for use. Transfer 100 μl of human whole blood into a labeled flow tube, mix the prepared antibody working solution well and add 10 μl to the whole blood. Gently vortex to mix, and incubate in the dark at 4°C for 30 minutes. After incubation, add 2 ml of red blood cell lysing solution to the flow tube and immediately vortex to mix. Lysis at room temperature for 10 minutes, observe if the liquid clarifies, and if clear, centrifuge at 1000 rpm for 5 minutes. Remove the flow tube from the centrifuge, discard the liquid in the tube to a waste bottle, and gently invert on paper to dry the tube opening. Add 2 ml of 1× phosphate buffer solution (PBS) for washing, centrifuge at 1500 rpm for 5 minutes, remove the supernatant, then add 200 μl of 1×PBS to resuspend, mix well before testing. After washing, the samples were immediately analyzed using BD FACSverse. Detailed information on the categorization of T, B, and NK cell labels is provided in **Supplementary Tables 1, 2**. Moreover, we provide representative flow cytometry plots demonstrating how we identified and analyzed specific cell populations (**Supplementary Figures 1–3**).

### 2.4 Development and assessment of prediction model

To select candidates for lymphocyte immunophenotyping, orthogonal partial least squares–discriminant analysis (OPLS-DA) was performed to explore discrimination among lymphocyte subtypes. An S-plot was constructed to visualize the contribution of lymphocyte subtypes to group discrimination. Variable importance in projection (VIP) values for each lymphocyte subtype were calculated to indicate their contributions to sample classification. Variables with VIP values greater than 1 were considered significant for discriminating the groups. Lymphocyte immunophenotyping candidates (VIP > 1 and P < 0.05) and significant clinical parameters (P < 0.05) were subjected to least absolute shrinkage and selection operator (LASSO) regression. Correlation coefficients between factors were then calculated, and a predictive scoring formula was established based on the expression levels of each factor multiplied by their corresponding regression coefficients. The predictive efficacy of the model and other lymphocyte immunophenotyping candidates was evaluated using receiver operating characteristic (ROC) curves. Diagnostic parameters, including AUC, sensitivity, and specificity, were determined using the “pROC” package. A nomogram was constructed to estimate the probability of recurrence based on the risk score and other factors. All identified biomarkers were then validated using random forest with 10-fold cross-validation, implemented through the R package “caret”.

### 2.5 Statistical analysis

All statistical analyses were applied by RStudio (v.4.2.0) and its corresponding packages as described previously. The significant statistic of general clinical characteristics analyzed by SPSS software was set at two-tailed and *P*<0.05. Continuous variables are presented as mean ± standard deviation and were analyzed using Student’s t-test or the Wilcoxon test. Categorical variables are expressed as frequencies and proportions and were analyzed using the Chi-square test or Fisher’s exact test. PCA, PLS-DA, OPLS-DA, and S-plot analyses were performed using SIMCA 13 (Umetrics). To further examine differences among these selected subsets across multiple groups, we applied an appropriate multiple comparison correction to control for potential Type I error inflation. An ANOVA followed by *post-hoc* Bonferroni or Kruskal–Wallis test with Dunn’s correction was used for *post-hoc* multiple comparisons. Correlation analysis was conducted using the “corrplot” package. The ‘Mfuzz’ package was employed to associate expression patterns of immunophenotypes with the progression of gout.

## 3 Results

### 3.1 Enrollment of participants and alteration of lymphocyte immunophenotyping

This study evaluated 165 patients diagnosed with gout who were admitted to the hospital between March 2022 and March 2023. After excluding 24 patients, we enrolled a total of 141 participants, comprising 78 individuals with acute gout (AG) and 63 patients in gout remission (RG) (**Supplementary Figure 4**). Additionally, we recruited 66 healthy volunteers as normal controls (NC). The baseline clinical characteristics of these participants are detailed in **Table 1**. There were no significant differences in age, height, and blood urea nitrogen (BUN) levels among the three groups. However, significant differences were observed in white blood cell (WBC) counts, platelet (PLT) counts, neutrophils, lymphocytes, eosinophils, basophils, and estimated glomerular filtration rate (eGFR) between patients during gout attacks and those in remission. Between patients in RG and NC, significant differences emerged in clinical indicators such as body mass index (BMI), red blood cell (RBC) counts, hemoglobin (Hb), monocytes, eGFR, and serum uric acid (UA) level.

**Table 1:** Clinical features among gout remission (RG), acute gout (AG) and normal control (NC) groups.

Variables	NC (n = 66)	RG (n = 63)	AG (n = 78)	P value^#^	*P* value^*^
Age (years)	45.50 (42.00,51.00)	50.00 (39.00,7.50)	46.50 (34.00,58.00)	0.142	0.378
Height (cm)	172.00 (170.00,175.75)	174.00 (170.00,178.00)	173.74 ± 5.26	0.298	0.995
Weight (kg)	74.00 (67.25,78.00)	77.00 (73.00,85.50)	78.00 (71.00,85.00)	**<0.001**	0.678
BMI (kg/m^2^)	24.54 (23.15,25.88)	25.96 (24.57,27.97)	25.67 (24.29,27.74)	**<0.001**	0.563
RBC (10^12^/L)	5.08 (4.79,5.32)	4.83 (4.47,5.29)	4.94 (4.58,5.30)	**0.016**	0.417
Hb (g/L)	156.00 (151.00,160.75)	148.00 (139.50,157.50)	154.00 (142.00,160.75)	**<0.001**	0.216
WBC (10^9^/L)	6.03 (5.34,7.02)	6.36 (5.65,7.37)	7.56 (6.61,9.22)	0.317	**<0.001**
PLT (10^9^/L)	246.64 ± 50.70	227.36 ± 57.44	240.50 (207.25,305.00)	**0.046**	**0.008**
N (%)	57.92 ± 7.40	58.61 ± 8.96	64.64 ± 9.53	0.635	**<0.001**
L (%)	32.83 ± 6.72	30.96 ± 9.61	25.95 ± 7.80	0.203	**0.001**
M (%)	5.85 (5.12,6.77)	6.60 (5.75,7.80)	6.40 (5.60,8.15)	**<0.001**	0.426
E (%)	2.20 (1.30,3.25)	2.10 (1.40,3.35)	1.80 (0.92,2.60)	0.927	**0.038**
B (%)	0.40 (0.20,0.60)	0.50 (0.30,0.75)	0.30 (0.20,0.50)	0.128	**<0.001**
BUN (mmol/L)	4.95 (4.10,5.30)	5.00 (4.15,6.30)	4.84 (4.21,5.91)	0.345	0.841
eGFR (mL/min/1.73m²)	102.45 (94.80,106.97)	92.66 (73.51,106.80)	77.80 (62.30,94.79)	**0.003**	**0.013**
SCr (µmol/L)	79.50 (71.25,85.00)	86.00 (74.00,101.50)	87.00 (79.12,104.50)	**0.002**	0.258
UA (μmol/L)	370.00 (334.25,398.00)	503.00 (407.50,577.00)	518.90 (452.75,571.55)	**<0.001**	0.475

^#^Comparison between NC and RG; ^*^Comparison between AG and RG.

Continuous variables following a normal distribution are described using the mean ± standard deviation; for those not following a normal distribution, the median (first quartile, third quartile) is used. BMI, body mass index; RBC, red blood cell; Hb, Hemoglobin; WBC, White Blood Cell; PLT, Platelets; N%, percentage of Neutrophil; L%, percentage of Lymphocytes; M%, percentage of Monocytes; E%, percentage of Eosinophils; B%, percentage of Basophils; BUN, Urea nitrogen; eGFR, estimated glomerular filtration rate; SCr, serum creatinine; UA, uric acid.

Bold value denotes statistically significant values (p < 0.05).

**Figure 1:**
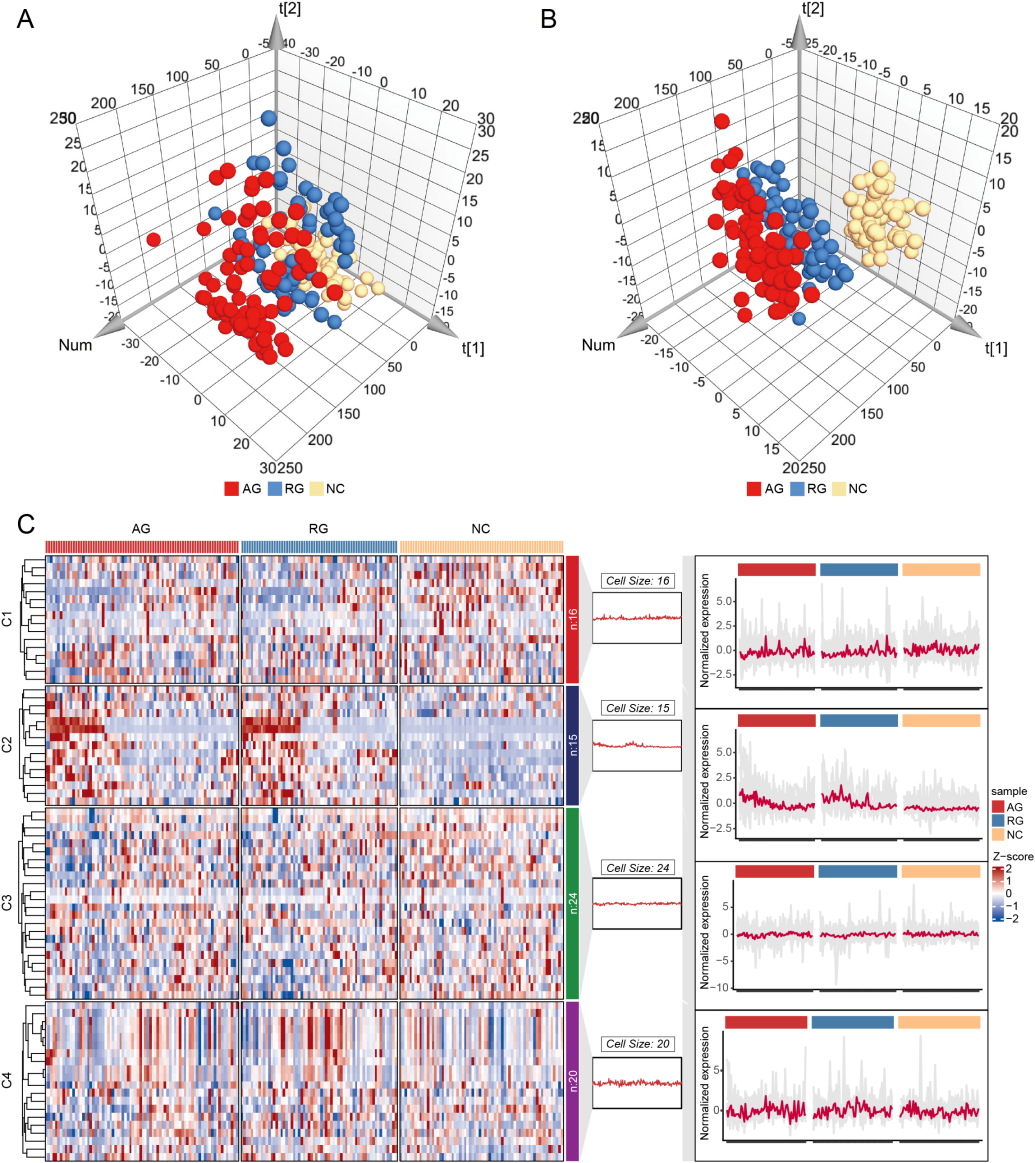
Principal component analysis (PCA) and Partial least squares-discriminant analysis (PLS-DA) for gout remission (RG), acute gout (AG) and normal control (NC) groups. **(A)** PCA for RG, AG and NC groups. **(B)** PLS-DA for RG, AG and NC groups. **(C)** Cluster analysis of the trend for lymphocyte immunophenotypes.

To investigate the distribution and function of lymphocyte subsets among NC, AG, and RG groups, we measured a total of 75 immunophenotypes using flow cytometry. Principal component analysis (PCA) and partial least squares discriminant analysis (PLS-DA) revealed differences in lymphocyte subset profiles among AG, RG, and NC groups, indicating a shift in adaptive immunity associated with gout status (**Figures 1A, B**). To further elucidate the lymphocyte immunophenotypes associated with gout progression, we ordered the samples according to the three states: gout flare, gout remission, and healthy controls. We divided the samples into four clusters using “Mfuzz” analysis, revealing a relatively stable cluster 3 and a highly reactive cluster 4 (**Figure 1C**). Interestingly, we found that with gout remission, cluster 2 gradually stabilized to levels seen in healthy individuals. This evidence suggests that lymphocyte subtypes play a crucial role in these significant shifts in gout patterns.

### 3.2 Lymphocyte subsets as complete gout remission diagnostic markers

**Figure 2:**
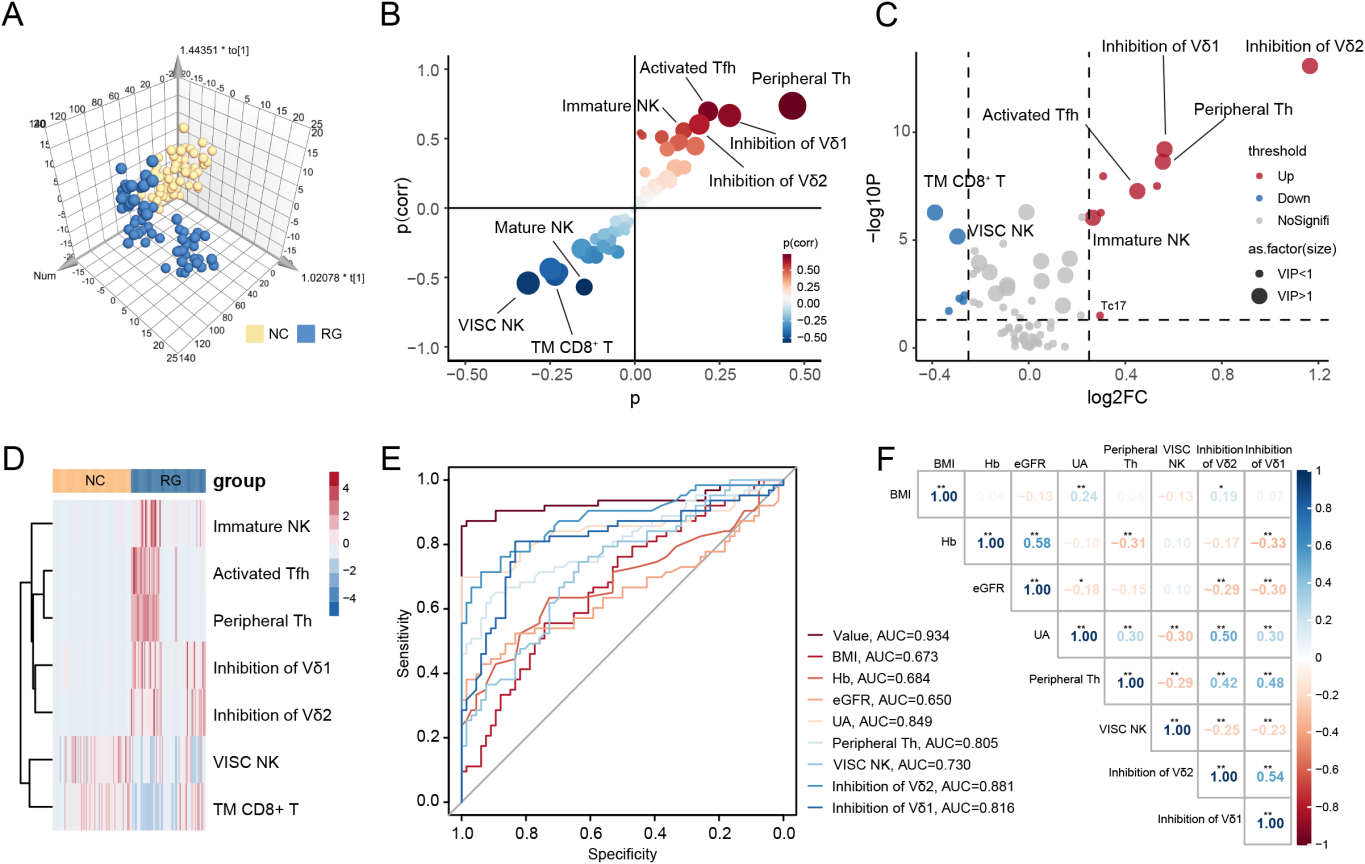
Screen diagnostic markers for complete gout remission. **(A)** Orthogonal partial least squares-discriminant analysis (OPLS-DA) for gout remission (RG) and normal control (NC) groups. **(B)** S-plot analysis of OPLS-DA. **(C)** The volcano plot represents the relative expression levels of lymphocyte subsets between RG and NC groups. **(D)** Heatmaps of selected lymphocyte subsets between RG and NC groups. All displayed lymphocyte subsets are statistically significant at the *P*<0.05 and VIP >1. **(E)** ROC curves comparing the predictive accuracy of the value and other factors. **(F)** Pearson correlation analysis was performed to delineate the relationship between predictive factors in RG patients. TM CD8^+^ T, Total Memory CD8^+^ T cells; VISC NK, Virus-Infected Specific Cytotoxic NK cell; BMI, body mass index; Hb, Hemoglobin; eGFR, estimated glomerular filtration rate; UA, uric acid; Th, T helper cells. The P-values are denoted as *P < 0.05, **P < 0.01.

To determine the immunological differences between RG and NC, we conducted pairwise comparisons between the two groups. Significant alterations were observed in 38 lymphocyte subsets when comparing NC with RG (**Supplementary Table 3**). A 3D scatter plot for the OPLS-DA showed distinct discrimination of RG patients from the NC (**Figure 2A**, **Supplementary Figure 5A**). S-plot analysis (**Figure 2B**) indicated that T peripheral helper (Tph) cells, activated follicular helper T (Tfh) cells, immature NK cells, inhibition of Vδ1 cells, Inhibition of Vδ2 cells, virus-infected specific cytotoxic NK cells, conventional killer NK cells, mature NK cells, total memory CD8^+^ T cells, and T cytotoxic (Tc) 17 cells contribute greatly to the discrimination of the two group. Variables with VIP value >1.0, *P* value <0.05, and log2|Fold change|>0.25 were considered to be potential differential lymphocytes subsets (**Figure 2C**). Finally, 7 lymphocytes subsets including Tph cells, activated Tfh cells, immature NK cells, inhibition of Vδ1 cells, inhibition of Vδ2 cells, virus-infected specific cytotoxic NK cells and total memory CD8^+^ T cells was selected (**Figure 2D**).

To further assess the potential diagnostic value of these lymphocyte subsets, we performed LASSO regression models on the previously identified differential clinical data and lymphocyte subsets (**Supplementary Figure 5B**). We calculated the correlation regression coefficients between indices and established a diagnostic scoring formula based on the level of each index multiplied by its corresponding regression coefficient. For distinguishing RG from NC, four lymphocyte subsets (Tph cells, virus-infected specific cytotoxic NK cells, inhibition of Vδ1 cells and inhibition of Vδ2 cells) and four clinical data (BMI, Hb, eGFR and UA) achieved a higher area under the curve (AUC) of 0.934 (**Figure 2E**).

We further evaluated whether the selected lymphocyte subsets in RG correlated with relevant clinical indices. As shown in **Figure 2F**, we observed that UA levels were positively correlated with the inhibition of Vδ2 cells (**Supplementary Figure 5C**). This finding suggests that inhibition of Vδ2 cells is associated with complete remission of gout, indicating that a single uric acid measurement may not adequately reflect the microenvironment in gout remission.

### 3.3 Nomogram development to predict the complete recovery of gout remission

**Figure 3:**
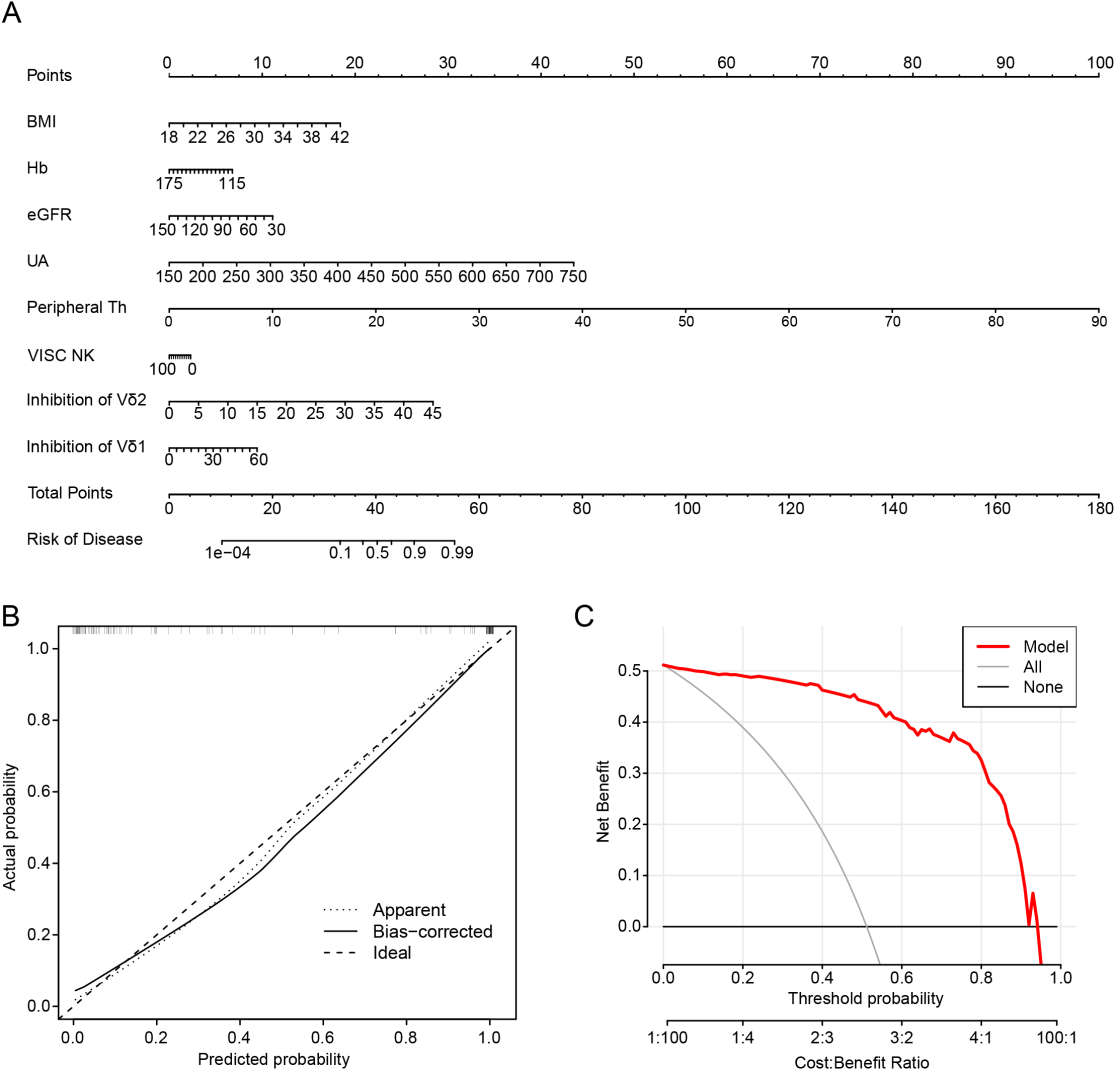
Nomogram development to predict the complete recovery of gout remission. **(A)** Nomogram in predicting complete remission of gout patients. **(B)** Calibration plots are used to evaluate the prediction effectiveness of nomogram. **(C)** Decision curve analysis (DCA) showed that making use of the nomogram for predicting the probability of gout remission. BMI, body mass index; Hb, Hemoglobin; eGFR, estimated glomerular filtration rate; UA, uric acid; Th, T helper cells; VISC NK, Virus-Infected Specific Cytotoxic NK cell.

Based on the results of LASSO regression analyses, we further constructed a nomogram by combining variables including lymphocyte subsets and clinical data (**Figure 3A**). The value at the intersection points represents the likelihood of an individual being in RG. Calibration plots assessed the predictive effectiveness of the nomogram (**Figure 3B**), and the calibration curve closely approximated a line with a slope of one, indicating strong concordance between predicted and actual values. Furthermore, decision curve analysis (DCA) demonstrated that utilizing the nomogram to predict the probability of gout remission yields greater net benefit, suggesting significant potential for clinical application (**Figure 3C**).

We found that controlling uric acid levels remains an essential indicator for achieving complete remission of gout. Moreover, alterations in the quantity of Tph cells and the inhibition of Vδ2 cells in the peripheral blood immune microenvironment are likely decisive factors in the disease trajectory toward complete remission.

### 3.4 Lymphocyte subsets as recurrent gout flare diagnostic markers

**Figure 4:**
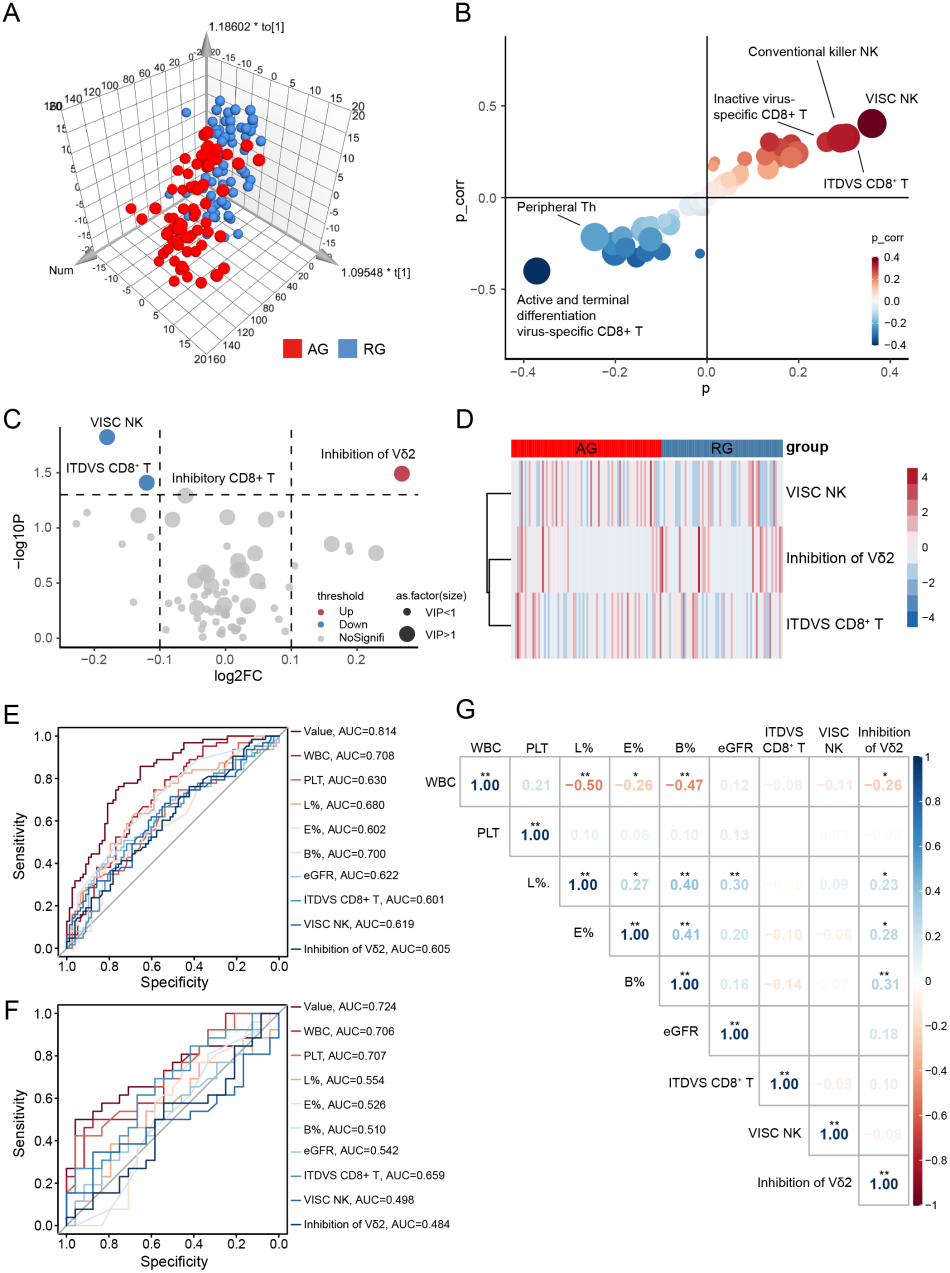
Screen diagnostic markers for recurrence gout. **(A)** Orthogonal partial least squares-discriminant analysis (OPLS-DA) for gout remission (RG) and acute gout **(AG)** groups. **(B)** S-plot analysis of OPLS-DA. **(C)** The volcano plot represents the relative expression levels of lymphocyte subsets between RG and AG groups. **(D)** Heatmaps of selected lymphocyte subsets between RG and AG groups. All displayed lymphocyte subsets are statistically significant at the P<0.05 and VIP >1. **(E)** AUC of ROC curves comparing the predictive accuracy of the value and other factors in training cohort. **(F)** ROC curves comparing the predictive accuracy of the value and other factors in validation cohort. **(G)** Pearson correlation analysis was performed to delineate the relationship between predictive factors in AG patients. VISC NK, Virus-Infected Specific Cytotoxic NK cell; ITDVS CD8^+^ T, Inactive and Terminal Differentiation Virus-Specific CD8^+^ T cells; WBC, white blood cell; PLT, platelets; L%, The percent of lymphocytes in peripheral blood; E%, The percent of Eosinophils in peripheral blood; B%, The percent of Basophils in peripheral blood. The P-values are denoted as *P < 0.05, **P < 0.01.

To assess the likelihood of recurrent attacks in gout patients in remission, we analyzed the distribution of peripheral blood lymphocytes. Significant changes were observed in three lymphocyte subsets when comparing AG with RG (**Supplementary Table 3**). In the OPLS-DA analysis, AG patients were distinctly separated from RG patients, illustrating evident differences in their peripheral blood lymphocyte profiles (**Figure 4A**, **Supplementary Figure 6A**). S-plot analysis and volcano plots indicated that inhibition of Vδ2 cells, virus-specific cytotoxic NK cells, and inactive and terminally differentiated virus-specific CD8^+^ T cells contributed significantly to the discrimination between the two groups (**Figures 4B–D**). Additionally, clinical indices such as WBC, PLT, and neutrophils—markers of inflammation—were significantly elevated in AG. Conversely, lymphocytes, eosinophils, basophils, and eGFR were increased in RG (**Table 1**).

Similarly, we sought diagnostic markers that could predict gout attacks in patients in remission using the previously described LASSO regression model (**Supplementary Figure 6B**). Ultimately, three lymphocyte subsets (inhibition of Vδ2 cells, virus-infected specific cytotoxic NK cells and inactive and terminal differentiation virus-specific CD8^+^ T cells) and six clinical parameters (WBC, PLT, lymphocytes, eosinophils, basophils and eGFR) were selected to perform ROC curve which achieved an AUC of 0.814 (**Figure 4E**). To further validate the reliability of the model, we conducted a one-year follow-up on patients in gout remission to observe potential recurrence of gout flare within a year. Remarkably, this model also achieved an AUC value of 0.724 in the validation cohort (**Figure 4F**).

We further evaluated whether the selected lymphocyte subsets in AG correlated with relevant clinical indices. As illustrated in **Figure 4G**, inhibition of Vδ2 cells exhibited extensive and close associations with various types of WBCs (**Supplementary Figure 6C**). This finding suggests that inhibition of Vδ2 cells may be implicated in the mechanisms underlying the recurrence of gout from remission.

### 3.5 Nomogram development to predict the possibility recurrence of gout flare

**Figure 5:**
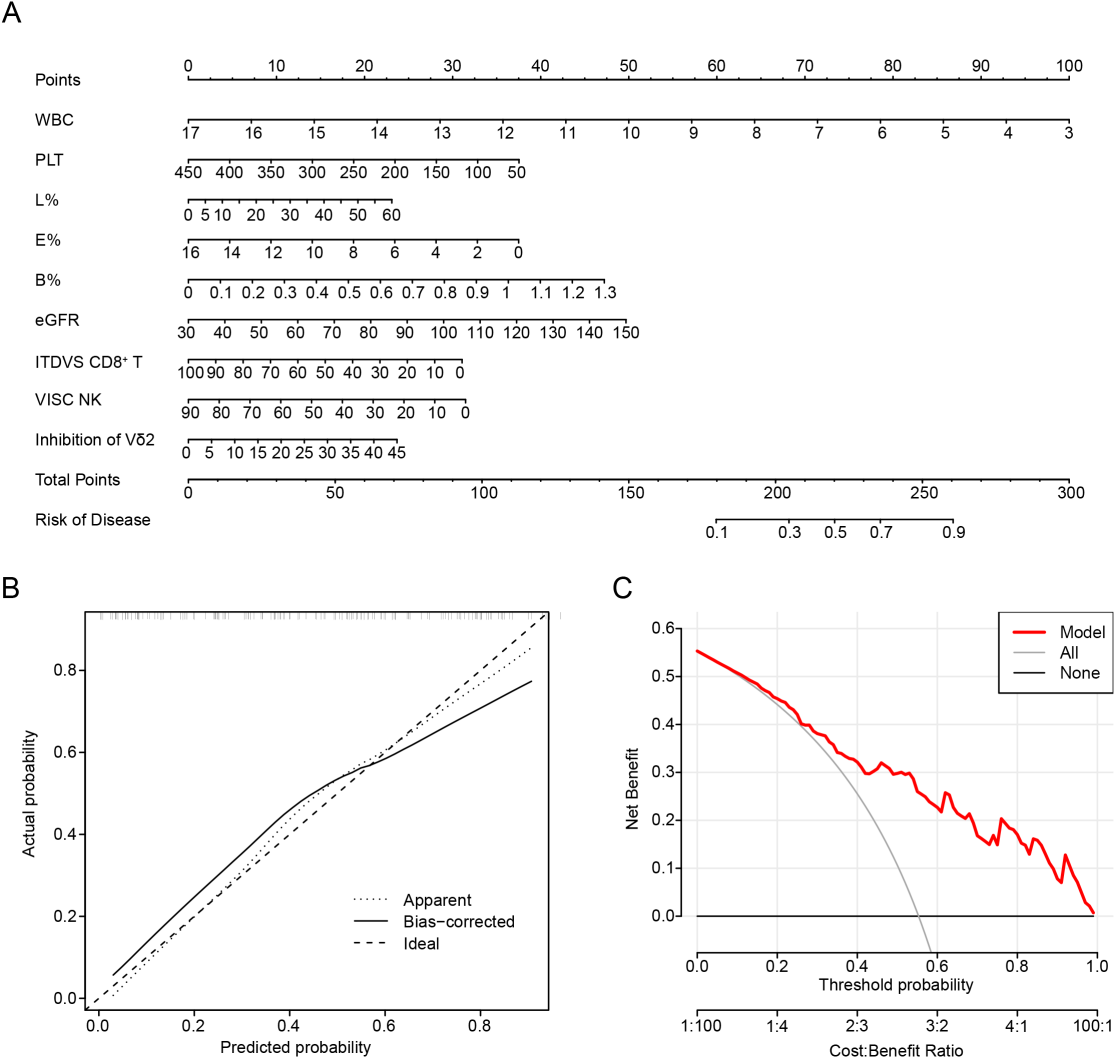
Nomogram development to predict the recurrence of gout. **(A)** Nomogram in predicting the recurrence of gout. **(B)** Calibration plots are used to evaluate the prediction effectiveness of nomogram. **(C)** Decision curve analysis (DCA) showed that making use of the nomogram for predicting the probability of recurrence of gout. VISC NK, Virus-Infected Specific Cytotoxic NK cell; ITDVS CD8^+^ T, Inactive and Terminal Differentiation Virus-Specific CD8^+^ T cells; WBC, white blood cell; PLT, platelets; L%, The percent of lymphocytes in peripheral blood; E%, The percent of Eosinophils in peripheral blood; B%, The percent of Basophils in peripheral blood.

Similarly, we constructed a nomogram by integrating lymphocyte subsets and clinical data (**Figure 5A**). The value corresponding to the intersection points represents the likelihood of an individual experiencing gout flares. Calibration plots were used to evaluate the predictive effectiveness of the nomogram (**Figure 5B**). The calibration curve closely aligned with a line of slope one, indicating strong concordance between predicted and actual values. Additionally, DCA showed that employing the nomogram to predict the probability of recurrence in gout remission provides greater net benefit, suggesting significant potential for clinical application (**Figure 5C**).

Through this nomogram, we understand that preventing gout recurrence necessitates continuous monitoring of white blood cell levels and the aforementioned immune cells. Furthermore, we propose that the level of eGFR is also an important indicator, suggesting that preserving renal function is beneficial in preventing gout recurrence.

### 3.6 Lymphocytes and clinical data exhibiting trend variations among the three groups

**Figure 6:**
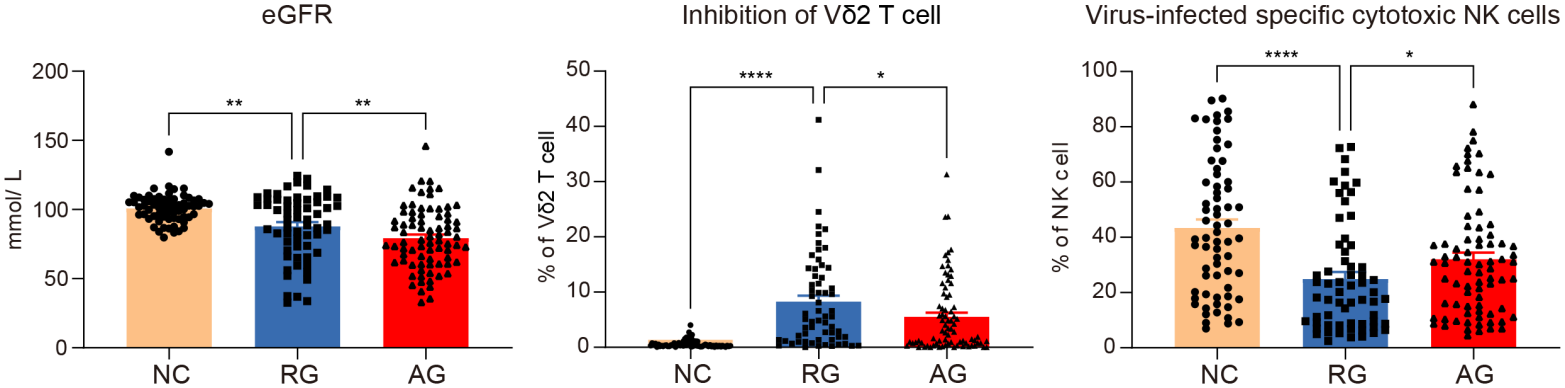
Significant variations of lymphocytes and clinical data among the gout remission (RG), acute gout (AG) and normal control (NC) groups. The P-values are denoted as *P* < 0.05, **P < 0.01 and **** *P* < 0.0001.

Based on our analysis, certain lymphocyte subsets exhibit significant differences among all groups. We found that eGFR showed trend variations across the groups, being lowest in the AG group and highest in the NC group. Additionally, we observed notable differences in the inhibition of Vδ2 cells and virus-specific cytotoxic NK cells, with these two cell types displaying inverse trends (**Figure 6**). Specifically, inhibition of Vδ2 cells was lowest in the NC group and highest in the RG group, whereas virus-specific cytotoxic NK cells were highest in the NC group and lowest in the RG group. Interestingly, for every 1% increase in the inhibition of Vδ2 cells, the adjusted relative risk ratio for complete remission of gout decreased by 37%. Each 1% increase in virus-specific cytotoxic NK cells was associated with a 1.3% greater adjusted relative risk ratio for complete remission of gout. Moreover, each 1% increase in virus-specific cytotoxic NK cells was associated with a 0.3% greater adjusted relative risk ratio for gout recurrence (**Tables 2**, **3**).

**Table 2:** Threshold effect analysis of immune cells on complete relief in gout remission.

	Per 1% increment	*P* value
Virus-infected specific cytotoxic NK cells	1.013 (1.006,1.020)	<0.001
Peripheral Th cells	0.860 (0.777, 0.929)	0.002
Inhibition of Vδ1 cells	0.926 (0.901, 0.949)	<0.001
Inhibition of Vδ2 cells	0.628 (0.537,0.715)	<0.001

Values are adjusted RR (95% CI). Model is adjusted for age (years), BMI (kg/m^2^). Th, T helper cells.

**Table 3:** Threshold effect analysis of immune cells on recurrence in gout remission.

	Per 1% increment	*P* value
Virus-infected specific cytotoxic NK cells	1.003 (1.000,1.005)	0.045
Terminal differentiation virus-specific CD8^+^ TC	1.002 (1.000,1.004)	0.077
Inhibition of Vδ2 cells	0.993 (0.986,1.000)	0.057

Values are adjusted RR (95% CI). Model is adjusted for age (years), BMI (kg/m^2^). TC, T cells.

## 4 Discussion

Gout arthritis typically resolves within 1-3 weeks following an attack, presenting as a self-limiting process, which is known to be associated with the activation and deactivation of synovial macrophages and neutrophils. However, the potential involvement of blood lymphocytes and their subsets in the phases of gout attack, complete remission, and recurrence is not explored. In this prospective cohort study, we initially profiled 75 lymphocyte immunophenotypes in populations experiencing gout flare, remission, and in healthy individuals. Strikingly, these lymphocyte subtypes displayed unique patterns during the transitions from gout flare to complete remission, and potentially, to recurrence. Furthermore, we discovered that the lymphocyte subtypes from cluster2 were characterized by immune inhibition and exhaustion (17) (**Supplementary Table 4**). Consequently, these findings underscore the pivotal role that circulating lymphocyte subtypes play in the distinct transformations associated with gout patterns.

The combined prediction of 4 types of immune cells and 4 clinical indicators achieved an impressive AUC value of 0.934 for complete remission. Notably, using UA values for individual prediction already achieves an AUC value of 0.849, indicating that lowering uric acid remains a crucial strategy for achieving complete remission in patients with RG (18, 19). Moreover, we discovered that utilizing clinical indicators alone or lymphocyte subtypes alone to differentiate between gout remission and healthy individuals resulted in AUC values of only 0.908 and 0.929, respectively. However, combining both increased the AUC value to 0.934 and enhanced specificity (**Supplementary Table 5**). Additionally, the construction of a predictive model between gout flare and remission further underscored the critical role of immune cells in forecasting gout recurrence, achieving an AUC of 0.814 in the training cohort and 0.724 in the testing cohort after one year of follow-up. The AUC values were only 0.797 and 0.651 when using clinical indicators alone or circulating lymphocyte subtypes alone, respectively. Combining both increased the AUC to 0.814 and also improved specificity (**Supplementary Table 5**). Therefore, these data suggests that specific circulating lymphocytes serve as reliable indicators for predicting gout transitions, presenting opportunities for early intervention and improved management of gout patients.

Our research demonstrated the inhibition of Vδ2 cells and virus-infected specific cytotoxic NK cells showed a potential mechanism in transitions between gout flare, complete remission, and recurrence. For every 1% increase in inhibition of Vδ1 cells and inhibition of Vδ2 cells, the adjusted relative risk for complete remission of gout was reduced by 7.4% and 37.2%, respectively (**Table 2**). We also similarly found that for every 1% increase in virus-infected specific cytotoxic NK cells, the relative risk of a patient having recurrent gout attacks increased by 0.3% (**Table 3**). Virus-infected specific cytotoxic NK cells are identified by Natural Cytotoxicity Triggering Receptor 1 (NKP46)^+^ expression which are related with activation of NK cell and secretion of interferon (IFN)-γ and tumor necrosis factor (TNF)-α (20, 21). In healthy individuals, high expression of these cells may monitor disease development and maintain health (22). Vδ2 cells, a subset of γδT cells, are crucial for the body’s innate immune surveillance, responding early to infections and stressed cells (23–25). During gout flare, the body mounts an inflammatory response against MSU crystals. Slightly increased inhibition of Vδ2 cells could help manage inflammation and clear MSU crystals, preventing excessive tissue damage or chronic inflammation. The highest inhibition of Vδ2 cells during gout remission might indicate a downregulated immune response to prevent overreaction to low levels of MSU crystals, helping maintain remission and prevent recurrence. Thus, these data indicates that inhibition of Vδ2 cells and virus-infected specific cytotoxic NK cells serve as reliable biomarkers for forecasting gout progression.

The 2018 EULAR evidence-based recommendations for the diagnosis of gout delineate its natural course into stages: asymptomatic hyperuricemia, acute flare, intercritical period, and chronic tophaceous gout (26). Notably, the 2019 edition of the “Chinese Guidelines for the Diagnosis and Treatment of Hyperuricemia and Gout” introduced the concept of subclinical gout, recognizing that some patients with asymptomatic hyperuricemia or in remission may exhibit MSU deposits in joint cavities, which can also result in corresponding health risks (27). Increasing evidence demonstrates that gout is a continuous pathological process and a recurrent chronic disease (28, 29). However, current staging criteria are insufficient in predicting gout recurrence and achieving complete remission. Although the criteria for complete remission were proposed in 2016, a subsequent study using dual-source CT confirmed the presence of MSU deposits in patients who met these criteria (6, 7). Other studies based on inflammatory markers in predicting gout flares also showed limited predictive value (30, 31). Traditional diagnostic standards primarily rely on clinical symptoms and laboratory markers, but these indicators do not fully reflect the immune status and disease progression of patients. Therefore, for the first time, this study proposes predicting gout flares and complete remission through dynamic changes in peripheral blood lymphocytes, providing valuable insights into the immunological staging of gout.

Our circulating lymphocyte immunophenotyping offers a more comprehension of the immune system’s dynamic alterations, facilitating the identification of distinct cell subsets implicated at different stages of disease progression and contributing to the elucidation of molecular mechanisms underlying inflammation initiation, maintenance, and resolution. Additionally, the study employed a prospective patient enrollment strategy, and the constructed model underwent independent sample validation, thereby affirming the robustness of our findings. However, there are some limitations. First, as a single-center study with a predominantly male cohort, our findings may not be fully generalizable to patients of different ethnicities, genders, or ages, future multi-center investigations with broader demographic representation are warranted; Second, we did not collect detailed lifestyle information (e.g., diet, exercise, alcohol consumption), which may influence immune responses in gout and could have impacted our results. In our future studies, we will plan to incorporate more comprehensive lifestyle assessments—such as validated dietary questionnaires, detailed physical activity logs, and alcohol consumption histories. Finally, this study is the lack of paired samples from the same patient during flare and remission periods for comparison.

Overall, our clinical data reveal significant shifts in lymphocyte immunophenotypes during the transitions among gout flare, complete remission, and recurrence. Variations in the inhibition of Vδ2 cells and virus-infected specific cytotoxic NK cells emerge as crucial immune phenotypes influencing gout recurrence and complete remission. The innovative model demonstrates substantial predictive value for the non-invasive diagnosis of both gout recurrence and complete remission. The two panels of combined models based on blood immune cells and clinical indicators offer substantial evidence for refining the clinical staging of gout and advancing the understanding of the mechanisms driving the transitions between gout flares, complete remission, and recurrence.

## Data Availability

The original contributions presented in the study are included in the article/**Supplementary Material**. Further inquiries can be directed to the corresponding authors.
