# Supplementary material for: Delineating circulating lymphocyte subsets in the transition from gout remission to recurrence
Source: Front Immunol. 2025 Apr 17;16:1540429. doi: 10.3389/fimmu.2025.1540429 (PMC12043595; doi:10.3389/fimmu.2025.1540429)
Supplement: Supplementary file 1 [file DataSheet1.docx]

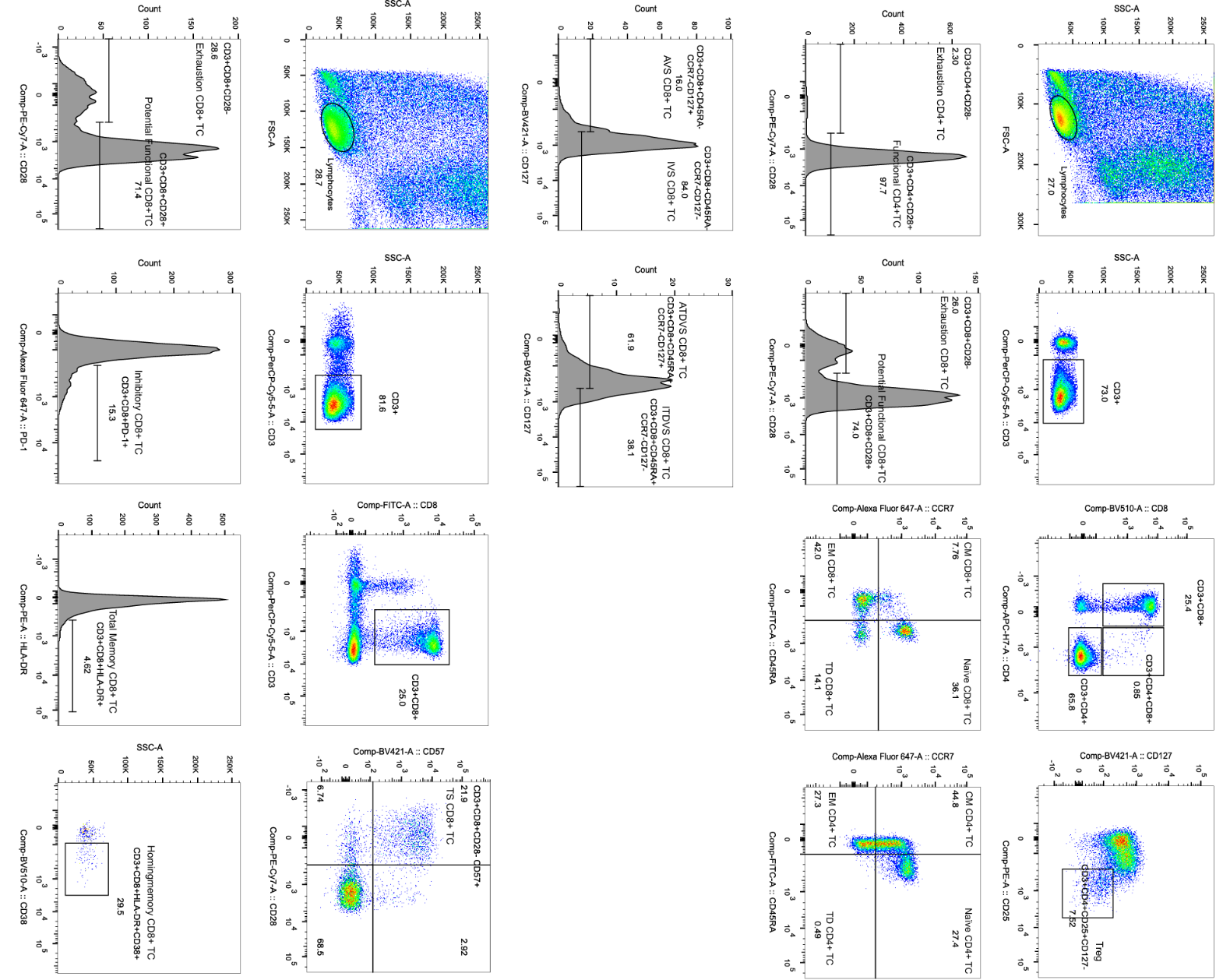


**Supplementary Figure 1 The flow cytometric gating strategy for T cell subsets.** TC: T cells; TD: Terminal Differential; CM: Central Memory; EM: Effective Memory; Treg: T regulatory; IVS: Inactive Virus-Specific; AVS: Active Virus-Specific; ITDVS: Inactive and Terminal Differentiation Virus-Specific; ATDVS: Active and Terminal Differentiation Virus-Specific; PF: Potential Functional; TM: Total Memory; TS: Terminally senescent;


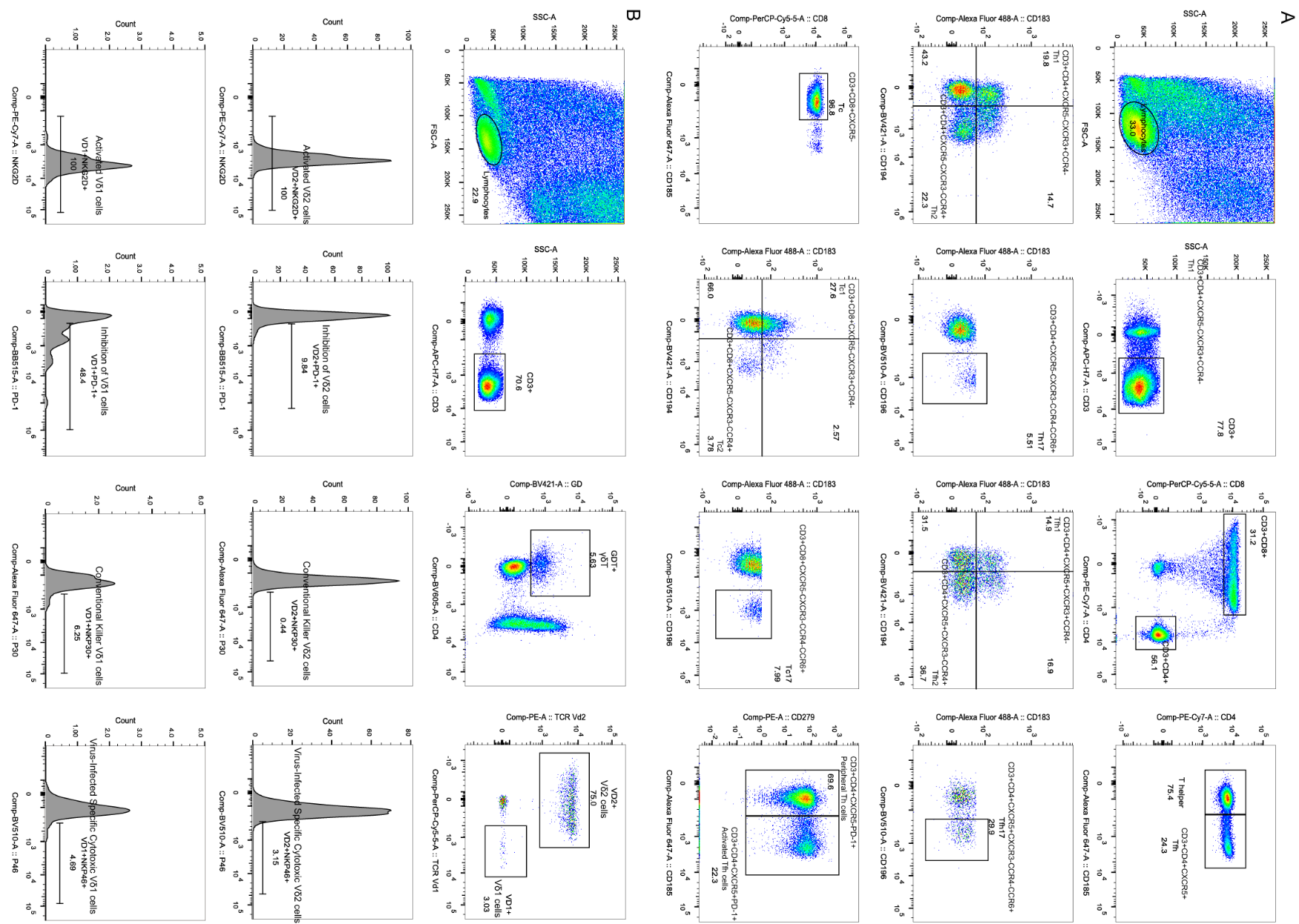


**Supplementary Figure 2 The flow cytometric gating strategy for Th cells, Tfh cells, Tc cells and γδT cells subsets.** (A) The gating strategy for Th cells, Tfh cells and Tc cells subsets. (B) The gating strategy for γδT cells subsets. TC: T cells; Th: T helper; Tc: T cytotoxic; Tfh: T follicular helper.


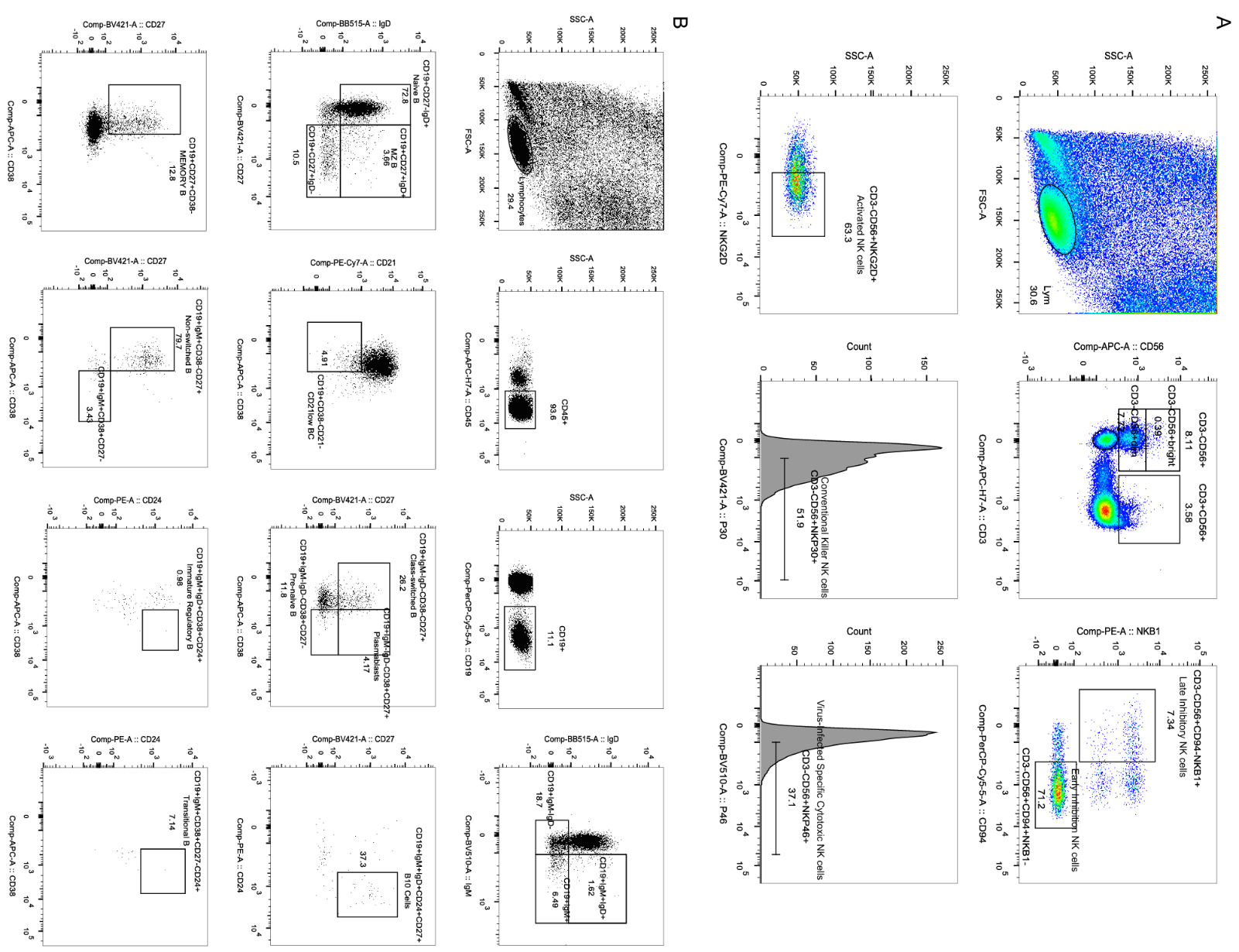


**Supplementary Figure 3 The flow cytometric gating strategy for NK cells and B cells subsets.** (A) The gating strategy for NK cells subsets. (B) The gating strategy for B cells subsets. NK: Nature killer; MZ: Marginal Zone.


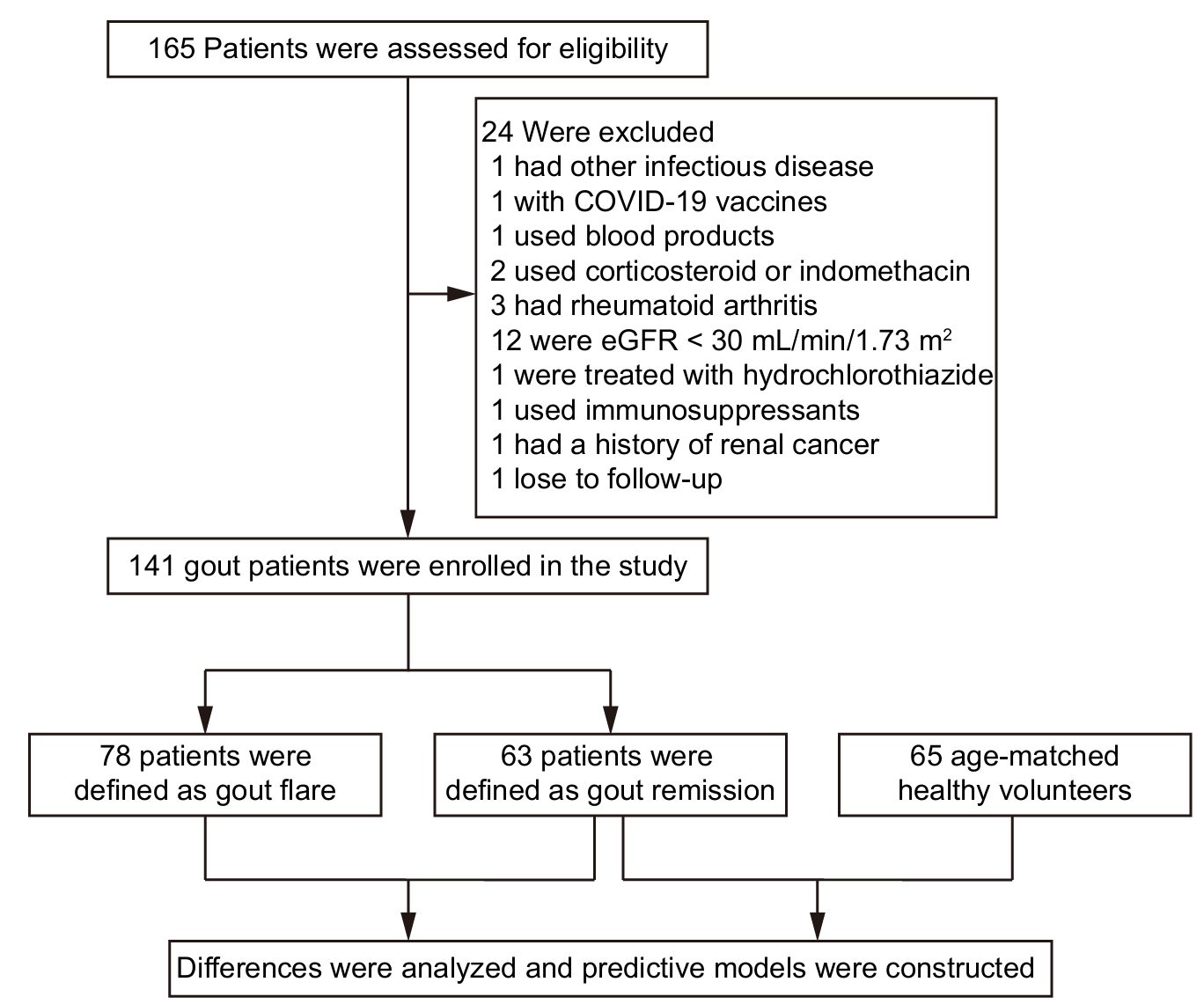


**Supplementary Figure 4 An overview of patient screening, patient assignment in this prospective study.**


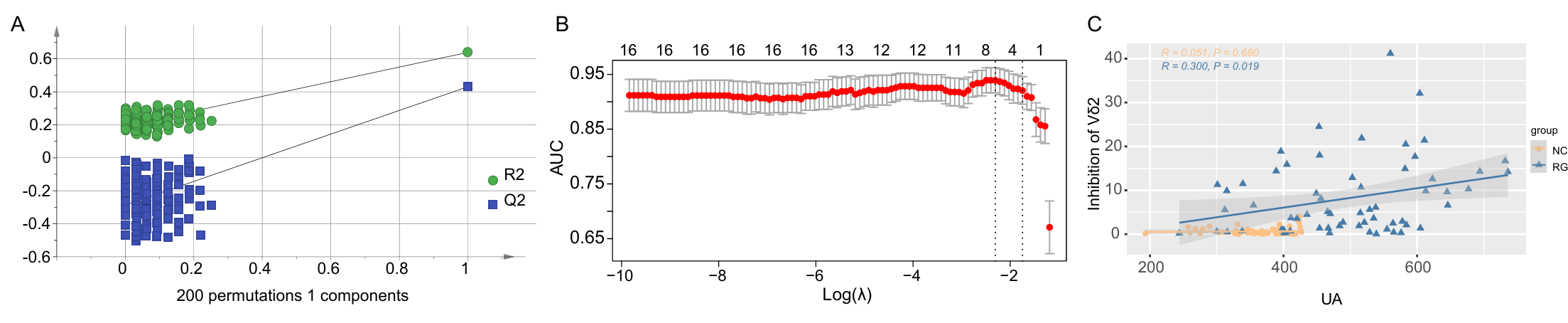


**Supplementary Figure 5 Lymphocyte subsets as complete gout remission diagnostic markers.** (A) The permutations plots for the gout remission patients and healthy individual all show the OPLS-DA models used above are valid. The green dots represent R2 while blue dots represent Q2. The dash lines represent the corresponding regression lines consistent with the dots color. The number of permutations for each plot was 200; (B) Optimal parameter (λ) selected in the LASSO Cox regression model based on the minimum criteria; (C) Pearson correlation analysis shows inhibition of Vδ2 cells positively associated with UA (Uric acid) in gout remission patients.


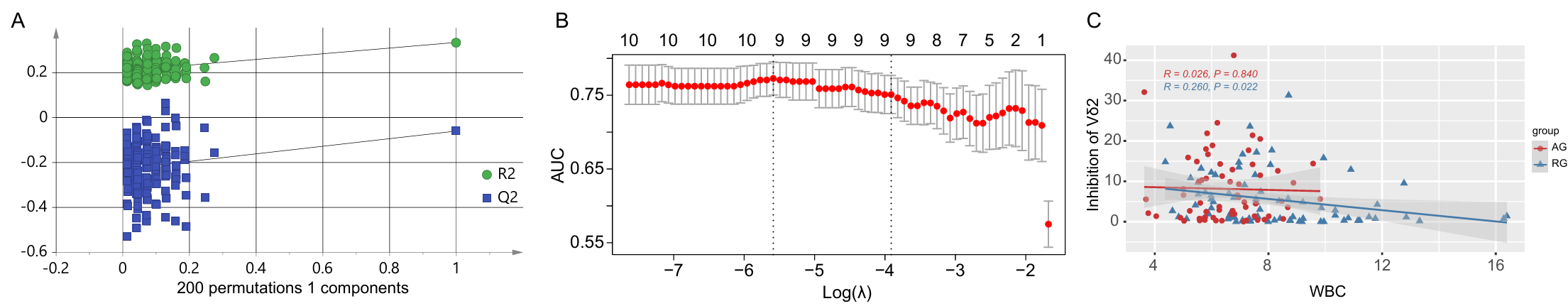


**Supplementary Figure 6 Lymphocyte subsets as recurrent gout flare diagnostic markers.** (A) The permutations plots for the gout flare and gout remission patients all show the OPLS-DA models used above are valid. The green dots represent R2 while blue dots represent Q2. The dash lines represent the corresponding regression lines consistent with the dots color. The number of permutations for each plot was 200; (B) Optimal parameter (λ) selected in the LASSO Cox regression model based on the minimum criteria; (C) Pearson correlation analysis shows inhibition of Vδ2 cells negatively associated with WBC (White blood cell) in gout remission patients.
