# Supplementary material for: Delineating circulating lymphocyte subsets in the transition from gout remission to recurrence
Source: Front Immunol. 2025 Apr 17;16:1540429. doi: 10.3389/fimmu.2025.1540429 (PMC12043595; doi:10.3389/fimmu.2025.1540429)
Supplement: Supplementary file 2 [file DataSheet2.docx]

**Supplementary Table 1 The information of antibody for flow cytometry**

| Antibody | Fluorescein | Cat No. | Source | Clone |
| --- | --- | --- | --- | --- |
| CD3 | APC-H7 | 560176 | BD | SK7 |
| CD4 | PE-Cy7 | 557852 | BD | SK3 |
| CD4 | APC-H7 | 560158 | BD | RPA-T4 |
| CD8 | PerCP-Cy5.5 | 565310 | BD | SK1 |
| CD8 | FITC | 555634 | BD | HIT8a |
| CD8 | BV510 | 563919 | BD | SK1 |
| CD28 | PE-Cy7 | 560684 | BD | CD28.2 |
| CD127 | BV421 | 562436 | BD | HIL-7R-M21 |
| CCR7 | AF647 | 560816 | BD | 150503 |
| CD21 | PE-Cy7 | 561374 | BD | B-ly4 |
| CD24 | PE | 555428 | BD | ML5 |
| CD25 | PE | 555432 | BD | M-A251 |
| CD45RA | FITC | 555488 | BD | HI100 |
| CD57 | BV421 | 568894 | BD | NK-1 |
| CD38 | BV510 | 563251 | BD | HIT2 |
| CD38 | APC | 555462 | BD | HIT2 |
| CD45 | APC-H7 | 560178 | BD | 2D1 |
| CD19 | PerCP-Cy5.5 | 561295 | BD | HIB19 |
| CD27 | BV421 | 562513 | BD | M-T271 |
| IgD | BB515 | 565243 | BD | IA6-2 |
| IgM | BV510 | 563113 | BD | G20-127 |
| PD-1 | PE | 557946 | BD | MIH4 |
| PD-1 | BB515 | 564494 | BD | EH12.1 |
| PD-1 | AF647 | 560838 | BD | EH12.1 |
| CD56 | APC | 555518 | BD | B159 |
| CD94 | PerCP-Cy5.5 | 562361 | BD | HP-3D9 |
| NKG2D | PE-Cy7 | 562365 | BD | 1D11 |
| NKP30 | BV421 | 563385 | BD | p30-15 |
| NKP30 | AF647 | 558408 | BD | p30-15 |
| NKP46 | BV510 | 564064 | BD | 9E2/NKp46 |
| NKB1 | PE | 555967 | BD | DX9 |
| HLA-DR | PE | 555812 | BD | G46-6 |
| TCRγδ | BV421 | 744870 | BD | 11F2 |
| TCRVδ1 | percp-vio700 | 130-120-441 | Miltenyi Biotec | REA173 |
| TCRVδ2 | PE | 555739 | BD | B6 |
| CXCR5 | AF647 | 558113 | BD | RF8B2 |
| CXCR3 | AF488 | 558047 | BD | 1C6/CXCR3 |
| CCR4 | BV421 | 562579 | BD | 1G1 |
| CCR6 | BV510 | 563241 | BD | 11A9 |

**Supplementary Table 2 Information on markers of lymphocyte subsets.**

| Celltypes (markers) | Celltypes (markers) |
| --- | --- |
| TC (CD3^+^) | γδT (CD3^+^CD4^-^GDT^+^) |
| Th cell (CD3^+^CD4^+^) | Vδ1 cells (CD3^+^CD4^-^GDT^+^VD1^+^) |
| Tc cell (CD3^+^CD8^+^) | Vδ2 cells (CD3^+^CD4^-^GDT^+^VD2^+^) |
| Double positive TC (CD3^+^CD4^+^CD8^+^) | Activated Vδ2 cells (CD3^+^CD4^-^GDT^+^VD2^+^NKG2D^+^) |
| Naïve CD4^+^ TC (CD3^+^CD4^+^CD45RA^+^CCR7^+^) | Inhibition of Vδ2 cells (CD3^+^CD4^-^GDT^+^VD2^+^PD1^+^) |
| TD CD4^+^ TC (CD3^+^CD4^+^CD45RA^+^CCR7^-^) | CK Vδ2 cells (CD3^+^CD4^-^GDT^+^VD2^+^NKP30^+^) |
| CM CD4^+^ TC (CD3^+^CD4^+^CD45RA^-^CCR7^+^) | VISC Vδ2 cells (CD3^+^CD4^-^GDT^+^VD2^+^NKP46^+^) |
| EM CD4^+^ TC (CD3^+^CD4^+^CD45RA^-^CCR7) | Activated Vδ1 cells (CD3^+^CD4^-^GDT^+^VD1^+^NKG2D^+^) |
| Exhaustion CD4^+^ TC (CD3^+^CD4^+^CD28^-^) | Inhibition of Vδ1 cells (CD3^+^CD4^-^GDT^+^VD1^+^PD1^+^) |
| Functional CD4^+^TC (CD3^+^CD4^+^CD28^+^) | CK Vδ1 cells (CD3^+^CD4^-^GDT^+^VD1^+^NKP30^+^) |
| Treg cell (CD3^+^CD4^+^CD25^+^CD127^-^) | VISC Vδ1 cells (CD3^+^CD4^-^GDT^+^VD1^+^NKP46^+^) |
| Naïve CD8^+^ TC (CD3^+^CD8^+^CD45RA^+^CCR7^+^) | BC (CD3^-^CD19^+^) |
| TD CD8^+^ TC (CD3^+^CD8^+^CD45RA^+^CCR7^-^) | Naïve BC (CD3^-^CD19^+^CD27^-^IgD^+^) |
| CM CD8^+^ TC (CD3^+^CD8^+^CD45RA^-^CCR7^+^) | MZ BC (CD3^-^CD19^+^CD27^+^IgD^+^) |
| EM CD8^+^ TC (CD3^+^CD8^+^CD45RA^-^CCR7^-^) | CD21^-^ BC (CD3^-^CD19^+^CD38^-^CD21^-^) |
| IVS CD8^+^ TC (CD3^+^CD8^+^CD45RA^-^CCR7^-^CD127^hi^) | Pre-naïve BC (CD3^-^CD19^+^IgD^-^IgM^-^CD27^-^CD38^+^) |
| AVS CD8^+^ TC (CD3^+^CD8^+^CD45RA^-^CCR7^-^CD127^lo^) | Plasmablast (CD3^-^CD19^+^IgD^-^IgM^-^CD27^+^CD38^+^) |
| ITDVS CD8^+^ TC (CD3^+^CD8^+^CD45RA^+^CCR7^-^CD127^hi^) | CS BC (CD3^-^CD19^+^IgD^-^IgM^-^CD27^+^CD38^-^) |
| ATDVS CD8^+^ TC (CD3^+^CD8^+^CD45RA^+^CCR7^-^CD127^lo^) | B10 cell (CD3^-^CD19^+^IgD^+^IgM^+^CD24^+^CD27^+^) |
| Inhibitory CD8^+^ TC (CD3^+^CD8^+^PD-1^+^) | Memory BC (CD3^-^CD19^+^CD27^+^CD38^-^) |
| Exhaustion CD8^+^ TC (CD3^+^CD8^+^CD28^-^) | NS BC (CD3^-^CD19^+^IgM^+^CD27^+^CD38^-^) |
| PF CD8^+^ TC (CD3^+^CD8^+^CD28^+^) | IR BC (CD3^-^CD19^+^IgD^+^IgM^+^CD38^+^CD24^-^) |
| TM CD8^+^ TC (CD3^+^CD8^+^HLA-DR^+^) | Transitional BC (CD3^-^CD19^+^IgD^+^IgM^+^CD27^-^CD24^+^) |
| TS CD8^+^ TC (CD3^+^CD8^+^CD28^-^CD57^+^) | NKC (CD3^-^CD56^+^) |
| Homingmemory CD8^+^ TC (CD3^+^CD8^+^ HLA-DR^+^CD38^+^) | Immature NKC (CD3^-^CD56^+bright^) |
| Tfh1 cells (CD3^+^CD4^+^CXCR5^+^CXCR3^+^CCR4^-^) | Mature NKC (CD3^-^CD56^+dim^) |
| Tfh2 cells (CD3^+^CD4^+^CXCR5^+^CXCR3^-^CCR4^+^) | EI of NKC (CD3^-^CD56^+^CD94^+^NKB1^-^) |
| Tfh17 cells (CD3^+^CD4^+^CXCR5^+^CXCR3^-^CCR4^-^CCR6^+^) | LI NKC (CD3^-^CD56^+^CD94^-^NKB1^+^) |
| Th1 cells (CD3^+^CD4^+^CXCR5^-^CXCR3^+^CCR4^-^) | Activated NK cells (CD3^-^CD56^+^NKG2D^+^) |
| Th2 cells (CD3^+^CD4^+^CXCR5^-^CXCR3^-^CCR4^+^) | CK NKC (CD3^-^CD56^+^NKP30^+^) |
| Th17 cells (CD3^+^CD4^+^CXCR5^-^CXCR3^-^CCR4^-^CCR6^+^) | VISC NKC (CD3^-^CD56^+^NKP46^+^) |
| Tc1 (CD3^+^CD8^+^CXCR5^-^CXCR3^+^CCR4^-^) | Tfh cells (CD3^+^CD4^+^CXCR5^+^) |
| Tc2 (CD3^+^CD8^+^CXCR5^-^CXCR3^-^CCR4^+^) | Peripheral Th cells (CD3^+^CD4^+^CXCR5^-^PD-1^+^) |
| Tc17 (CD3^+^CD8^+^CXCR5^-^CXCR3^-^CCR4^-^CCR6^+^) | Activated Tfh cells (CD3^+^CD4^+^CXCR5^+^PD-1^+^) |

TC: T cells; Th: T helper; Tc: T cytotoxic; TD: Terminal Differential; CM: Central Memory; EM: Effective Memory; Treg: T regulatory; IVS: Inactive Virus-Specific; AVS: Active Virus-Specific; ITDVS: Inactive and Terminal Differentiation Virus-Specific; ATDVS: Active and Terminal Differentiation Virus-Specific; PF: Potential Functional;

TM: Total Memory; TS: Terminally senescent; Tfh: T follicular helper; CK: Conventional Killer; VISC: Virus-Infected Specific Cytotoxic; BC: B cell; MZ: Marginal Zone; CS: Class-Switched; NS: Non-Switched; IR: Immature Regulatory; NKC: Nature killer cell; EI: Early Inhibition; LI: Late Inhibitory.

**Supplementary Table 3 The alterations of 75 immunophenotypes among normal control (NC), gout remission (RG) and acute gout flare (AG).**

| Variables | NC (n = 66) | RG (n = 63) | AG (n = 78) | *P* value^#^ | *P* value^*^ |
| --- | --- | --- | --- | --- | --- |
| TC | 60.59±10.77 | 68.17±10.61 | 66.40(57.28,72.88) | <0.001 | 0.080 |
| Th cell | 54.64±11.36 | 61.77±12.16 | 59.15±11.84 | <0.001 | 0.200 |
| Tc cell | 32.76±9.79 | 29.84±11.02 | 31.83±9.80 | 0.115 | 0.265 |
| Th/Tc | 1.78(1.21,2.29) | 1.56(1.13,2.57) | 1.69 (1.28, 2.27) | 0.821 | 0.977 |
| Double positive TC | 2.16(1.54,3.19) | 1.78(1.08,3.00) | 1.72 (0.83, 2.61) | 0.127 | 0.438 |
| Naïve CD4^+^ TC | 40.49±13.19 | 40.94±17.68 | 36.92±16.62 | 0.869 | 0.170 |
| TD CD4^+^ TC | 1.87(1.17,4.98) | 2.02(1.04,4.20) | 1.98 (0.93, 4.18) | 0.519 | 0.797 |
| CM CD4^+^ TC | 32.97±9.33 | 28.85±10.79 | 31.09±12.41 | 0.022 | 0.252 |
| EM CD4^+^ TC | 19.25(15.33,26.80) | 23.70(16.30,29.40) | 22.35(16.73,32.60) | 0.117 | 0.697 |
| Exhausted CD4^+^ TC | 6.00 (2.23, 11.62) | 3.21(1.38,5.94) | 3.43(1.32,7.22) | 0.007 | 0.546 |
| Functional CD4^+^ TC | 94.00(88.38,97.77) | 96.80(94.05,98.60) | 96.55(92.75,98.68) | 0.007 | 0.543 |
| Treg cell | 5.23(4.30,6.29) | 4.10(2.84,5.06) | 3.90(2.96,4.78) | <0.001 | 0.701 |
| Naïve CD8^+^ TC | 12.35(7.46,20.92) | 17.10(11.25,32.25) | 16.40(7.49, 26.23) | 0.011 | 0.242 |
| TD CD8^+^ TC | 42.60(31.38,59.40) | 29.30(14.40,45.50) | 32.60(18.60,52.83) | <0.001 | 0.301 |
| CM CD8^+^ TC | 5.00(3.25,7.64) | 4.98 (3.18, 7.22) | 3.91(2.36,7.58) | 0.783 | 0.220 |
| EM CD8^+^ TC | 30.25(22.05,47.55) | 37.40(28.70,51.45) | 38.15(29.25,50.85) | 0.065 | 0.903 |
| IVS CD8^+^ TC | 63.35±19.30 | 59.92±18.45 | 65.60(45.73,79.05) | 0.304 | 0.335 |
| AVS CD8^+^ TC | 36.65±19.30 | 38.68±18.32 | 33.55(19.65,54.88) | 0.540 | 0.304 |
| ITDVS CD8^+^ TC | 29.45(19.88,41.20) | 25.60(16.05,38.60) | 33.75(19.97,50.48) | 0.220 | **0.039** |
| ATDVS CD8^+^ TC | 70.55(58.80,80.12) | 76.70(62.65,84.05) | 66.60(49.52,82.05) | 0.082 | 0.084 |
| Inhibitory CD8^+^ TC | 23.20(18.92,32.05) | 18.90(11.95,25.20) | 21.75(17.12,28.15) | 0.001 | 0.051 |
| Exhaustion CD8^+^ TC | 34.95(23.77,47.25) | 33.00(26.90,53.10) | 36.50(23.13,50.15) | 0.387 | 0.534 |
| PF CD8^+^ TC | 65.05(52.75,76.22) | 67.00(46.60,72.50) | 61.95(49.17,77.20) | 0.310 | 0.510 |
| TM CD8^+^ TC | 25.70(18.45,37.82) | 10.50(3.30,26.35) | 11.30(4.14,27.70) | <0.001 | 0.407 |
| Homingmemory CD8^+^ TC | 14.55(9.73,21.62) | 15.80(9.02,30.65) | 21.45(15.25,37.60) | 0.534 | 0.077 |
| TS CD8^+^ TC | 24.25(17.18,35.80) | 20.10(13.40,29.45) | 19.35(10.08,33.90) | 0.112 | 0.845 |
| Tfh | 18.16±5.33 | 18.30±5.78 | 18.50(14.43,22.30) | 0.882 | 0.576 |
| Tfh1 | 15.30(12.80,17.85) | 14.70(11.65,16.40) | 15.10(11.12,18.28) | 0.261 | 0.533 |
| Tfh2 | 35.90(31.55,40.03) | 35.10(30.00,40.75) | 35.25(29.40,41.18) | 0.536 | 0.611 |
| Tfh17 | 7.98(5.49,10.70) | 11.80(7.89,15.10) | 9.71(6.97,13.35) | <0.001 | 0.075 |
| Th1 | 21.05(15.88,26.28) | 15.40(11.00,21.95) | 17.20(11.43,23.25) | 0.003 | 0.420 |
| Th2 | 17.62±6.06 | 15.96±6.47 | 15.40(11.75,19.98) | 0.137 | 0.791 |
| Th17 | 0.88(0.54,1.51) | 1.79(1.23,3.05) | 1.73(0.94,2.89) | <0.001 | 0.234 |
| Th1/Th2 | 1.25(0.72,1.87) | 0.99(0.63,1.49) | 1.07(0.66,1.61) | 0.183 | 0.742 |
| Th17/Th2 | 0.05(0.03,0.10) | 0.17(0.07,0.24) | 0.11(0.07,0.21) | <0.001 | 0.163 |
| (Th1+Th17)/Th2 | 1.30(0.75,1.95) | 1.26(0.72,1.88) | 1.22(0.79,1.72) | 0.736 | 0.855 |
| Tc1 | 49.24±13.76 | 40.11±18.12 | 43.60(28.28,55.18) | 0.002 | 0.660 |
| Tc2 | 5.36(3.38,7.66) | 3.15(1.63,5.05) | 3.35(1.91,6.48) | <0.001 | 0.378 |
| Tc17 | 1.52(0.88,2.98) | 3.00(1.09,5.54) | 3.04(1.17,5.28) | 0.032 | 0.919 |
| Peripheral Th cells | 1.31(0.82,1.92) | 4.71(1.59,69.35) | 2.78(1.50,63.40) | <0.001 | 0.169 |
| Activated Tfh cells | 0.88(0.68,1.21) | 2.48(1.09,14.95) | 1.71(0.90,9.75) | <0.001 | 0.140 |
| γδT cell | 6.31(4.01,11.17) | 3.85(2.09,6.50) | 3.95(2.46,7.10) | <0.001 | 0.576 |
| Vδ1 cell | 16.4(8.11,38.02) | 16.90(6.96,35.15) | 16.20(7.69,31.10) | 0.749 | 0.821 |
| Vδ2 cell | 63.2(41.55,80.45) | 55.50(35.45,77.75) | 55.35(37.35,76.02) | 0.464 | 0.616 |
| Vδ1/Vδ2 | 0.27(0.10,0.92) | 0.34(0.10,0.96) | 0.32(0.11,0.82) | 0.631 | 0.983 |
| Activated Vδ2 cells | 99.60(98.82,99.90) | 99.80(99.00,100.00) | 99.65(98.90,100.00) | 0.092 | 0.313 |
| Inhibition of Vδ2 cells | 0.35(0.20,0.75) | 5.12(1.37,12.75) | 2.76(0.76,8.68) | <0.001 | **0.032** |
| CK Vδ2 cells | 0.45(0.22,0.90) | 0.21(0.00,0.98) | 0.29(0.02,0.79) | 0.019 | 0.471 |
| VISC Vδ2 cells | 0.34(0.21,0.80) | 0.48(0.11,1.34) | 0.81(0.34,1.70) | 0.869 | 0.092 |
| Activated Vδ1 cells | 98.50(97.53,99.40) | 99.30(97.00,100.00) | 98.80(96.10,99.60) | 0.101 | 0.147 |
| Inhibition of Vδ1 cells | 4.38(1.90,6.62) | 16.00(8.38,25.25) | 10.40(3.79,26.50) | <0.001 | 0.146 |
| CK Vδ1 cells | 1.92(0.86,3.68) | 1.04(0.00,2.95) | 1.27(0.32,2.60) | 0.004 | 0.482 |
| VISC Vδ1 cells | 1.67(1.01,3.16) | 1.04(0.12,2.51) | 1.69(0.66,3.92) | 0.009 | 0.073 |
| TNK cells | 5.81(3.60,8.88) | 3.47(1.99,5.52) | 2.88(2.03,7.24) | <0.001 | 0.674 |
| BC | 9.14 (7.41, 11.95) | 10.20 (7.18, 13.10) | 10.55 (8.38, 13.55) | 0.249 | 0.712 |
| Naïve BC | 58.05 (50.73, 67.38) | 59.40 (41.35, 68.60) | 59.65 (47.45, 68.92) | 0.710 | 0.498 |
| MZ BC | 11.85 (9.02, 17.55) | 10.90 (4.96, 15.75) | 9.19 (6.69, 16.05) | **0.041** | 0.534 |
| CD21- BC | 6.08 (4.38, 8.33) | 8.62 (6.00, 12.60) | 8.60 (4.96, 13.88) | **<0.001** | 0.609 |
| Pre-naïve BC | 0.20 (0.10, 0.36) | 0.22 (0.08, 0.44) | 0.26 (0.12, 0.50) | 0.612 | 0.455 |
| Plasmablast | 0.62 (0.30, 1.06) | 0.32 (0.10, 0.78) | 0.46 (0.17, 0.72) | **0.005** | 0.140 |
| CS BC | 20.94±8.29 | 20.45±10.91 | 17.00 (9.51, 25.02) | 0.776 | 0.239 |
| B10 cell | 0.42 (0.25, 0.70) | 0.69 (0.23, 1.30) | 0.56 (0.26, 1.31) | **0.031** | 0.867 |
| Memory BC | 33.25 (26.35, 41.45) | 29.10 (23.80, 40.95) | 31.65 (23.22, 41.88) | 0.414 | 0.824 |
| NS BC | 0.84 (0.52, 1.22) | 1.67 (0.94, 3.54) | 1.55 (0.71, 4.32) | **<0.001** | 0.560 |
| IR BC | 0.13 (0.07, 0.20) | 0.10 (0.04, 0.21) | 0.13 (0.06, 0.28) | 0.210 | 0.121 |
| Transitional BC | 0.05 (0.02, 0.11) | 0.06 (0.01, 0.12) | 0.07 (0.02, 0.15) | 0.857 | 0.530 |
| NKC | 15.05 (11.30, 20.15) | 11.80 (7.08, 17.75) | 12.15 (7.52, 18.62) | **0.013** | 0.402 |
| Immature NKC | 2.60 (1.83, 3.34) | 4.79 (2.68, 7.26) | 4.21 (2.71, 6.31) | **<0.001** | 0.335 |
| mature NK C | 97.40 (96.70, 98.18) | 95.20 (92.05, 97.30) | 95.70 (93.10, 97.30) | **<0.001** | 0.382 |
| Immature NKC / mature NKC | 0.03 (0.02, 0.03) | 0.05 (0.03, 0.08) | 0.04 (0.03, 0.07) | **<0.001** | 0.344 |
| EI NKC | 48.51±16.46 | 51.36±18.54 | 52.02±21.26 | 0.359 | 0.843 |
| LI NKC | 9.32 (5.28, 13.28) | 5.50 (3.44, 10.55) | 6.78 (3.10, 11.90) | **0.007** | 0.839 |
| Activated NKC | 87.55 (76.05, 90.97) | 83.40 (71.50, 92.55) | 83.70 (70.45, 92.00) | 0.478 | 0.606 |
| CK NKC | 50.30 (33.67, 65.12) | 31.30 (20.25, 48.65) | 37.70 (24.13, 56.60) | **<0.001** | 0.084 |
| VISC NKC | 39.70 (20.58, 59.98) | 20.10 (8.80, 33.60) | 30.45 (14.48, 40.52) | **<0.001** | **0.015** |

^#^Comparison between NC and RG; ^*^Comparison between AG and RG

Continuous variables following a normal distribution are described using the mean ± standard deviation; for those not following a normal distribution, the median (first quartile, third quartile) is used. TC: T cells; Th: T helper; Tc: T cytotoxic; TD: Terminal Differential; CM: Central Memory; EM: Effective Memory; Treg: T regulatory; IVS: Inactive Virus-Specific; AVS: Active Virus-Specific; ITDVS: Inactive and Terminal Differentiation Virus-Specific; ATDVS: Active and Terminal Differentiation Virus-Specific; PF: Potential Functional; TM: Total Memory; TS: Terminally senescent; Tfh: T follicular helper; CK: Conventional Killer; VISC: Virus-Infected Specific Cytotoxic; BC: B cell; MZ: Marginal Zone; CS: Class-Switched; NS: Non-Switched; IR: Immature Regulatory; NKC: Nature killer cell; EI: Early Inhibition; LI: Late Inhibitory.

**Supplementary Table 4 The lymphocyte subsets profile in Cluster2.**

| Cluster 2 immune cells subtypes | Marker |
| --- | --- |
| T cells | CD3^+^ |
| Effective memory CD4^+^ T cells | CD3^+^CD4^+^CD45RA^-^CCR7^-^ |
| Effector memory CD8^+^ T cells | CD3^+^CD8^+^CD45RA^-^CCR7^-^ |
| Terminally senescent CD8^+^ T cells | CD3^+^CD8^+^CD28^-^CD57^+^ |
| Tfh17 cells | CD3^+^CD4^+^CXCR5^+^CXCR3^-^CCR4^-^CCR6^+^ |
| Th17 cells | CD3^+^CD4^+^CXCR5^-^CXCR3^-^CCR4^-^CCR6^+^ |
| Peripheral Th cells | CD3^+^CD4^+^CXCR5^-^PD-1^+^ |
| Activated Tfh cells | CD3^+^CD4^+^CXCR5^+^PD-1^+^ |
| Inhibition of Vδ2 cells | CD3^+^CD4^-^GDT^+^VD2^+^PD1^+^ |
| Inhibition of Vδ1 cells | CD3^+^CD4^-^GDT^+^VD1^+^PD1^+^ |
| Immature NK cells | CD3^-^CD56^+bright^ |
| CD21^-^ B cells | CD3^-^CD19^+^CD38^-^CD21^-^ |
| Pre-naïve B cells | CD3^-^CD19^+^IgD^-^IgM^-^CD27^-^CD38^+^ |
| B10 cells | CD3^-^CD19^+^IgD^+^IgM^+^CD24^+^CD27^+^ |
| Non-switched B cells | CD3^-^CD19^+^IgM^+^CD27^+^CD38^-^ |

**Supplementary Table 5 ROC Curve Analysis in Diagnosis of gout complete remission and gout flare**

| Indicators | AUC (95% CI) | P value | Youden Index | Sensitivity (%) | Specificity (%) |
| --- | --- | --- | --- | --- | --- |
| Diagnosis of gout complete remission | | | | | |
| Model 1 | 0.934 (0.882, 0.986) | <0.001 | 0.860 | 0.873 | 0.955 |
| Model 2 | 0.908 (0.856, 0.959) | <0.001 | 0.735 | 0.794 | 0.939 |
| Model 3 | 0.929 (0.880, 0.978) | <0.001 | 0.814 | 0.873 | 0.939 |
| Diagnosis of gout flare | | | | | |
| Model 4 | 0.814 (0.745, 0.883) | <0.001 | 0.515 | 0.753 | 0.762 |
| Model 5 | 0.797 (0.856, 0.959) | <0.001 | 0.499 | 0.769 | 0.730 |
| Model 6 | 0.651 (0.560, 0.741) | 0.001 | 0.274 | 0.551 | 0.730 |

Model 1: BMI + Hb + eGFR + UA + Tph + virus-infected specific cytotoxic NK cells + inhibition of Vδ1 +inhibition of Vδ2;

Model 2: BMI + Hb + eGFR + UA;

Model 3: Tph + virus-infected specific cytotoxic NK cells + inhibition of Vδ1 +inhibition of Vδ2;

Model 4: WBC + PLT + L% + E% + B% + eGFR + Terminal differentiation virus-specific CD8^+^ T cells + Virus-infected specific cytotoxic NK cells + Inhibition of Vδ2;

Model 5: WBC+PLT+L%+E%+B%+eGFR;

Model 6: Terminal differentiation virus-specific CD8^+^ T cells + Virus-infected specific cytotoxic NK cells + Inhibition of Vδ2;

BMI, body mass index; HB, hemoglobin; eGFR, estimated glomerular filtration rate; UA, uric acid; Tph, T peripheral helper; WBC, white blood cell; PLT, platelet; L%, percentage of lymphocytes; E%, percentage of eosinophils; B%, percentage of basophils; eGFR, estimated glomerular filtration rate
